# A Fourier Transform Spectrometer Based on an Electrothermal MEMS Mirror with Improved Linear Scan Range

**DOI:** 10.3390/s16101611

**Published:** 2016-09-29

**Authors:** Wei Wang, Jiapin Chen, Aleksandar. S. Zivkovic, Huikai Xie

**Affiliations:** 1 Department of Micro-Nano Electronics, Shanghai Jiao Tong University, Shanghai 200240, China; chenjp@sjtu.edu.cn; 2 Department of Electrical and Computer Engineering, University of Florida, Gainesville, FL 32611, USA; lacasner@ufl.edu (A.S.Z.); hkxie@ece.ufl.edu (H.X.)

**Keywords:** Fourier transform spectrometer, microelectromechanical systems (MEMS), electrothermal micromirror, closed-loop control

## Abstract

A Fourier transform spectrometer (FTS) that incorporates a closed-loop controlled, electrothermally actuated microelectromechanical systems (MEMS) micromirror is proposed and experimentally verified. The scan range and the tilting angle of the mirror plate are the two critical parameters for MEMS-based FTS. In this work, the MEMS mirror with a footprint of 4.3 mm × 3.1 mm is based on a modified lateral-shift-free (LSF) bimorph actuator design with large piston and reduced tilting. Combined with a position-sensitive device (PSD) for tilt angle sensing, the feedback controlled MEMS mirror generates a 430 µm stable linear piston scan with the mirror plate tilting angle less than ±0.002°. The usable piston scan range is increased to 78% of the MEMS mirror’s full scan capability, and a spectral resolution of 0.55 nm at 531.9 nm wavelength, has been achieved. It is a significant improvement compared to the prior work.

## 1. Introduction

Fourier transform spectroscopy is a well-established technique that has been used to determine a variety of unknown substances with applications ranging from chemical and biomedical sensing to hazardous materials detection. Fourier transform spectrometers (FTS) have the advantages of large spectral range covering thousands of wavenumbers, high signal-to-noise ratio (SNR), and high spectral resolution compared to the dispersive spectrometers [1], but conventional FTSes are table-top systems, which are bulky and expensive and typically are for lab use only. To realize real-time monitoring, online process control, and on-site detection, miniaturized FTS systems must be developed.

A typical FTS system is mainly based on Michelson interferometer, which has a scanning mirror and a fixed mirror and uses a single photodetector to pick up the temporal interferogram signal. Similarly, spatial heterodyne spectrometers (SHS) are also based on Michelson interferometer but without moving parts. However, SHS require photodetector arrays and have serious trade-offs between spectral resolution and spectral bandwidth [2]. So, in this work, we just focus on the classic Michelson interferometer based FTS systems. The mirror scan range in an FTS determines its spectral resolution; the larger the mirror scan range, the higher the spectral resolution. Thus, one of the major tasks for FTS miniaturization is to reduce the size of the mirror scanning mechanism without seriously sacrificing the spectral performance.

Microelectromechanical systems (MEMS) technology is a very powerful tool for miniaturization, and MEMS micromirrors have been developed for a wide range of applications, such as projectors [3,4,5], optical switching [6], endoscopic optical coherence tomography (OCT) [7,8,9], microscopy [10], and spectroscopy [11,12]. Electrostatic, electromagnetic, piezoelectric, and electrothermal actuation mechanisms are typically employed in MEMS mirrors. The choice of the type of actuation mechanism depends on the actual application by considering the required range, speed, and voltage. For example, the most popular electrostatic MEMS mirror is Texas Instruments’ (Dallas, TX, USA) digital micromirror device (DMD) [3,4], which has been successfully commercialized for portable projectors and projection displays for nearly two decades. Electromagnetic MEMS mirrors are employed in laser scanning displays due to their high speed and large scan range [5,13]. Piezoelectric deformable MEMS mirrors have been used in adaptive optics systems for their precise displacement control [14,15]. Electrothermal MEMS mirrors have been applied in endoscopic OCT imaging due to their large scan range, high fill factor, and low voltage [8,9].

MEMS mirror-based FTS spectrometers have been reported by many research groups in recent years [16,17,18,19,20], and their main difference lies in the actuation mechanism for generating the piston motion. K. Yu et al. reported a micromachined FTS based on an electrostatic comb-drive micro mirror with the scan range of 25 µm at high voltage of 150 V [16]. T. Sandner et al. proposed another electrostatic micromirror based on a pantograph lever design capable of a maximum 500 µm displacement, but it required high vacuum (50 Pa) and must operate at resonance [17]. U. Wallrabe et al. developed an FTS system based on an electromagnetic actuator by LIGA fabrication (Freiburg, Germany), in which the travel range of the mirror was 54 µm [18]. Besides, some piezoelectric mirrors for piston motion were also reported [19,20]. Qiu et al. presented a translational piezoelectric actuator with a maximum vertical displacement of about 86 µm at 20 V [21]. These MEMS mirrors have limited linear scan range of less than 100 µm. Even though operating at resonance in high vacuum could boost the scan range, but this creates issues such as large dynamic deformation and difficulty in implementing any mirror plate tilting compensation.

In contrast, electrothermally actuated MEMS mirrors can generate a large linear scan range up to 1 mm at low drive voltage without operating at resonance [22]. However, the issue of the mirror plate tilting that significantly limits the usable scan range is a bottleneck for electrothermal MEMS mirror-based FTS systems. Wu et al. developed a lateral-shift-free (LSF) electrothermal MEMS mirror with 620 μm vertical displacement at 5.3 V, but the mirror plate experienced a tilting as large as 0.7° [23]. Liu et al. proposed a piston MEMS with small tilting by employing a curled concentric electrothermal bimorph actuator (CCBA) design which generated a 200 μm piston range with <0.4° tilting at 0.9 V [24], while Samuelson et al. reported a laddered inverted-series-connected (ISC) electrothermal actuator, which showed a 0.25° tilt at 90 µm piston displacement [25]. In addition to improving the structural design, Wu et al. attempted to compensate the tilting of the LSF mirror down to 0.06° by optimizing the ratio of the driving voltages, but the usable range was only about 70 μm out of the full 1000 μm [26]. Wang et al. employed a meshed inverted-series-connected (ISC) bimorph mirror in an FTS and reduced the tilt angle down to 0.004° also using the ratioed voltage method, but only in a range of 48 μm [27]. Later, Wang et al. extended the usable scan range to 225 μm with the tilting reduced down to ±0.002° using an open-loop control method [28], but it still only utilized less than 40% of the full scan range of the MEMS mirror. Furthermore, open-loop control is very sensitive to any environmental disturbances and also degrades quickly over time. These electrothermally-actuated MEMS mirrors are summarized in Table 1 in terms of the scan range and tilt angle with and without compensation.

In this work, an FTS system based on a closed-loop controlled LSF electrothermal MEMS mirror is developed. This LSF electrothermal micromirror can generate a maximum vertical displacement of 550 μm. By employing a closed-loop control method, the usable scan range of the MEMS mirror reaches 430 μm with the tilting controlled within ±0.002°. This is 78% of the full scan range of the MEMS mirror. The performance of this closed-loop control micromirror is also listed in Table 1. A high spectral resolution of 19.4 cm^−1^, or 0.55 nm at 531.9 nm wavelength, is demonstrated in the FTS system, which is a significant improvement on both MEMS design and tilting compensation method compared to the prior work.

## 2. The Principle of Fourier Transform Spectrometer (FTS)

**Figure 1:**
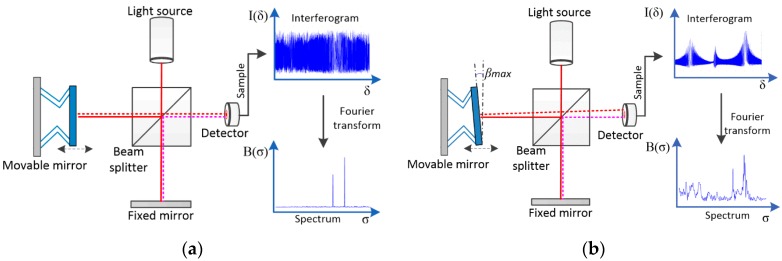
(**a**) An ideal FTS system; (**b**) An FTS system with the moving mirror tilting.

An FTS generates the spectrum of a source radiation by modulating the radiation in the time domain through interference, which is then Fourier transformed [29]. For a Michelson interferometer-based FTS system as shown in Figure 1a—which consists of a beam splitter, a photodetector, and two plane mirrors (one movable, one fixed) that are perpendicular to each other. The incident light is split by the beam splitter into two light beams that respectively reach the two mirrors. The two beams are then reflected off from the two mirrors and recombined at the beam splitter. The photodetector detects the optical power of the recombined beam that varies according to the relative position of the movable mirror. The zero optical path difference, or ZPD, is defined as the position of the movable mirror where the light beam reflected back from the movable mirror has the same optical path length as that from the fixed mirror does. At ZPD, the photodetector picks up the maximum optical power. As the movable mirror moves away from ZPD, the photodetector records a signal I(δ), which is also called interferogram, which encodes the spectral information of the light source, as a function of the optical path difference (OPD), δ. The spectrum B(σ) can be obtained by performing Fourier transform on the interferogram I(δ), as shown in Equation (1).

(1) $$B\left(\sigma \right)=\int_{-\infty }^{\infty }I\left(\delta \right)\exp\left(-i2\pi \sigma \delta \right)d\delta$$

where σ is the wavenumber (σ = 1/λ).

The theoretical finite resolution of the FTS is given by Equation (2),

(2) $$\Delta \sigma =1/\delta_{\max}$$

where $\delta_{\max}$ is the maximum OPD scan range, which is the double of the maximum displacement of the movable mirror.

The resolution of an FTS system is determined by the maximum of the mirror displacement, but it requires the planes of the mirrors remain in good alignment during the entire scanning. The tilting of the moving mirror plate is one of the primary sources of error in an FTS system. As shown in Figure 1b, the mirror plate tilting largely deteriorates the interferogram and leads to a reduction of the usable scan range which subsequently causes spectral resolution degradation [30]. Thus, in order to ensure minimal degradation of resolution, the maximum allowable tilt angle, β_max_, is given by [31]

(3) $$\beta_{\max}<\;\frac{1}{20\cdot D\cdot \nu_{\max}}$$

where *D* is the diameter of the light beam and ν_max_ is the wavenumber of the shortest-wavelength component of the light source under test. If *D* is 0.1 cm and ν_max_ is 15,800 cm^−1^, a tilt angle no more than 0.002° is desired for no significant resolution degradation. Therefore, when designing MEMS mirrors for FTS, we must consider not only the extension of the scan range, but also the compensation of the tilting effect.

## 3. MEMS Mirror Design & Fabrication

**Figure 2:**
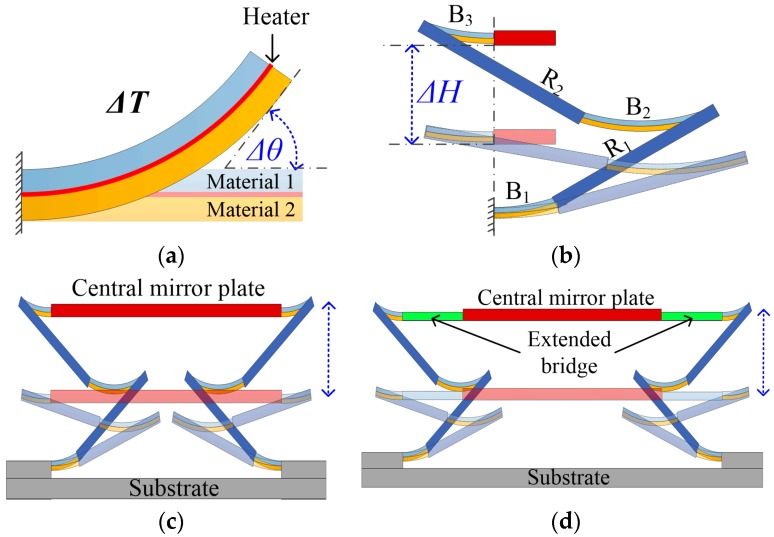
MEMS mirror design: (**a**) The bimorph structure; (**b**) The LSF actuator; (**c**) The basic schematic of LSF MEMS; (**d**) The LSF MEMS with extended bridge.

The actuation method employed in the movable micromirror of this FTS system is electrothermal bimorph actuation. As shown in Figure 2a, a bimorph consists of two materials with different coefficients of thermal expansion (CTEs) and an embedded resistor. The temperature of the bimorph changes via Joule heating, which results from injecting an electrical current to the resistor. The temperature change, *ΔT*, together with a non-zero CTE difference of the two bimorph materials, *Δ*α, generates thermal stresses that will bend the bimorph structure. The bending angle, *Δ*θ, at the tip of the bimorph is given by Equation (4) [31],

(4) $$\Delta \theta =\frac{\beta_{b}\cdot l}{t}\cdot \Delta \alpha \cdot \Delta T$$

where β*_b_* is the curvature coefficient, which is a constant determined by the properties of the bimorph materials, and *l* and *t* are respectively the length and thickness of the bimorph. For high actuation, aluminum (Al) and silicon dioxide (SiO_2_) are chosen as the bimorph materials for their large CTE difference [32].

A lateral-shift-free (LSF) electrothermal bimorph actuator design is employed for generating large piston displacement. The structure of the LSF bimorph actuator consists of two silicon-backed rigid beams (*R*_1_ and *R*_2_), and three Al/SiO_2_ bimorphs (*B*_1_, *B*_2_ and *B*_3_), as shown in Figure 2b. The lengths of *R*_1_ and *R*_2_, *L_R_*_1_ and *L_R_*_2_, are equal, and the lengths of the three bimorphs must satisfy the relation *L_B_*_2_ = *L_B_*_1_ + *L_B_*_3_. So, to the first order, the tilt and the lateral shift can be compensated, and the angular actuation of the Al/SiO_2_ bimorphs can be converted into a pure large vertical displacement, *ΔH*, which is expressed by Equation (5).

(5) $$\Delta H=\left(L_{R1}+L_{R2}\right)\cdot sin\left(\Delta \theta \right)$$

The LSF actuator-based MEMS mirror design is schematically shown in Figure 2c, where two LSF actuators, anchored on the substrate, support the central mirror plate on two sides symmetrically. There is a platinum (Pt) resistor embedded in each of the LSF actuators. When a voltage is applied to the Pt resistors in both actuators, the mirror plate moves vertically up the electrothermal actuation.

Due to process variations, the two actuators always have some small differences, resulting in a tilting of the mirror plate during its vertical scanning. When such a MEMS mirror is used as the movable mirror in an FTS system, the tilting effects will lead to spectral resolution degradation. The tilt angle, *Δ*γ*_t_*, of the mirror plate can be expressed as

(6) $$\Delta \gamma_{t}={tan}^{-1}\left(\frac{\Delta d}{a}\right)$$

where *Δd* is the vertical displacement difference between the two actuators, and *a* is the distance between the two actuators or the mirror plate length if the actuators are connected to the mirror plate directly. According to Equation (6), the larger the *a*, the smaller the tilt angle is. Based on the consideration of tilting issue mentioned above, an extended bridge separating the central mirror plate from the connection points of the LSF actuators—as shown in Figure 2d—is employed, instead of simply enlarging the mirror size which would increase the mass and reduce the resonance frequency of the device. As the result, the tilt angle can be reduced to some extended by this design.

**Figure 3:**
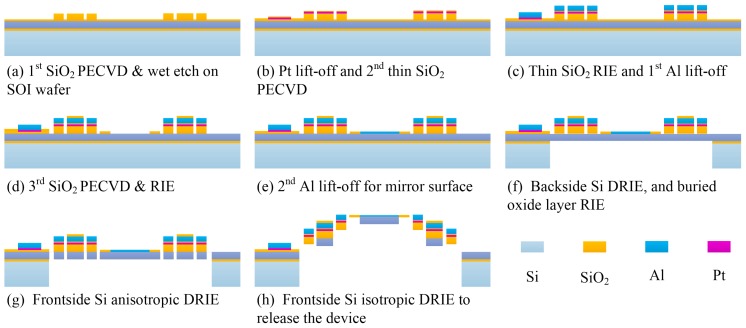
The fabrication process of the MEMS mirror.

The MEMS device is fabricated using a process flow combining bulk- and surface-micromachining on SOI wafers (device layer: 30 µm, handle layer: 300 µm, and buried oxide layer: 2 µm). It starts from the 1st SiO_2_ layer (1 µm thick) deposited with plasma enhanced chemical vapor deposition (PECVD) and patterned with buffered oxide etchant (BOE) (Figure 3a). Then a 150 nm-thick Pt is sputtered and lifted off to form the heater layer, followed by the second thin PECVD SiO_2_ layer (100 nm) deposited as the isolation (Figure 3b). Before the first Al layer (1 µm) is sputtered and lifted off, a reactive ion etch (RIE) of SiO_2_ is carried out to make the contact opening between the Pt heater and the Al pads and wiring (Figure 3c). Up to this step, both bimorph materials for the bimorph structures have been formed. After that, the third SiO_2_ layer (600 nm) is deposited with PECVD and patterned with RIE to define the bimorph actuators and also to serve as the Deep-RIE (DRIE) etching mask in the release step (Figure 3d). Then a second Al layer (200 nm) is deposited and patterned with lift-off to form the mirror surface (Figure 3e). Next, the process turns to the backside of the SOI wafer. A backside silicon DRIE is first performed to etch the silicon all way to the buried oxide (BOX) layer and then the BOX layer is removed by RIE to form the release cavity under the mirror plate (Figure 3f). Finally, a front-side silicon anisotropic DRIE is performed to etch through the device layer (Figure 3g), followed by isotropic DRIE to undercut the silicon underneath the Al/SiO_2_ bimorphs to complete the device release (Figure 3h).

**Figure 4:**
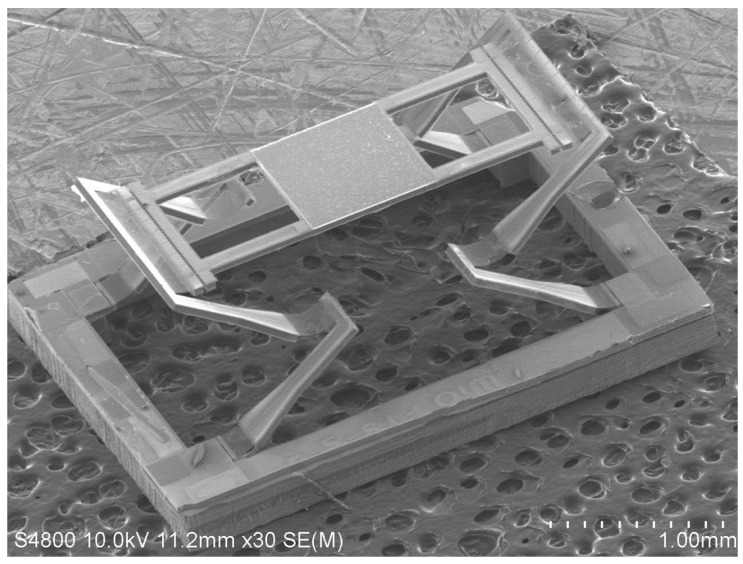
An SEM of a fabricated MEMS mirror.

Figure 4 shows an SEM of a fabricated device with a footprint of 4.3 mm × 3.1 mm. The structural parameters are listed in Table 2. The mirror plate is a silicon plate (the device layer of the SOI wafer) coated with aluminum for high reflectance, and it has an initial elevation of about 550 μm, which is caused by the intrinsic stresses and thermal residual stresses of the thin films in the bimorphs during the fabrication process.

**Figure 5:**
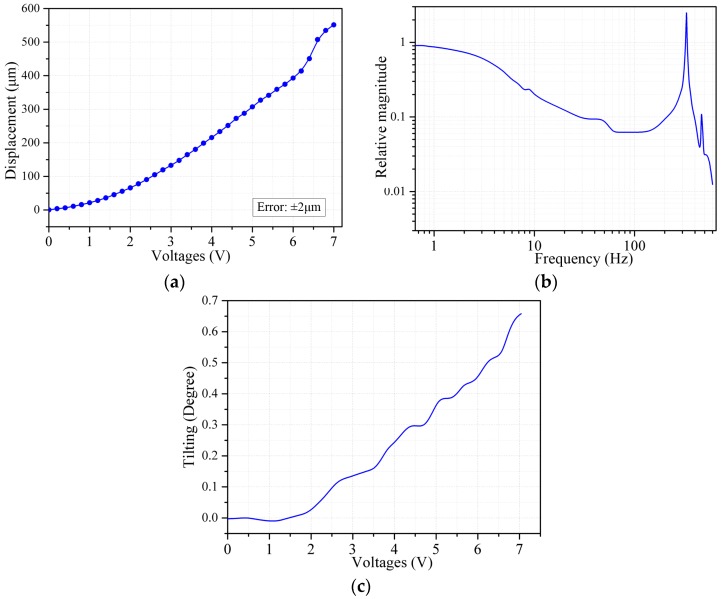
(**a**) Quasi-static response curve; (**b**) Frequency response curve; (**c**) The tilt angle versus voltage.

The measured quasi-static response and frequency response are shown in Figure 5a,b, respectively. The maximum vertical displacement reached 550 µm at 7 Vdc, and the measured first resonance frequency was 329 Hz. Based on the FEM simulation, the first resonance frequency would be decreased to 221 Hz if a full-filled, instead of bridged, mirror plate were used. Figure 3c shows the tilt angle upon actuation. The tilt angle measured at 3.5 V was 0.15°, which is a reduction by factor of 2, compared to 0.3° in our former work [28]. This is attributed to the extended bridge design. However, the maximum tilt angle of the mirror plate still reaches 0.65° under the drive voltage of 7 V, which is an obstacle to make the most use of the full scan range of the MEMS mirror.

## 4. Closed-Loop Control of the MEMS

The tilt shown in Figure 5c is directly caused by the small mismatches between the two bimorph actuators, such as resistance difference, bimorph layer thickness difference, beam width difference, and non-uniform stress distribution resulting from the inevitable process variations. Since the tilt angle becomes larger with the increasing voltage, the open-loop control method reported in [28] is only effective for the scan range up to 225 μm at 3.5 V. The tilt may be also influenced by environmental vibrations or airflow disturbance, which may interrupt the open-loop compensation. So a real-time closed-loop control method is introduced in this work to provide a more robust solution.

**Figure 6:**
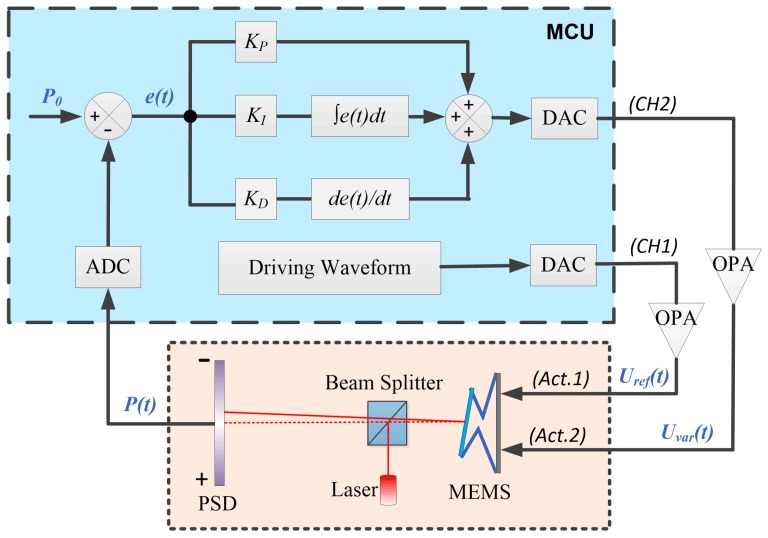
The block diagram of the closed-loop control. Note: MCU is micro control unit, ADC is analog to digital converter, DAC is digital to analog converter, OPA is operational amplifier, and PSD is position-sensitive device.

The block diagram of the closed-loop control is sketched in Figure 6. A micro control unit (MCU) is used for controlling and driving the MEMS mirror. A pre-determined voltage waveform is stored in the MCU and converted into an analog signal, *U_ref_*(*t*), which is applied to one of the actuators of the MEMS mirror, i.e., Act.1, leading to a vertical displacement and a tilt of the mirror plate. Via a beam splitter, a laser beam is directed to and reflected off the mirror surface. The laser beam continues to pass through the beam splitter and reach to a position-sensitive device (PSD). The PSD tracks the tilt of the mirror plate by locating the incident position of the laser spot. The output signal of the PSD, *P*(*t*), is digitized and compared with a preset voltage, *P*_0_, which corresponds to the target tilt angle. The difference between *P*(*t*) and *P*_0_, or the error, *e*(*t*), is fed to a PID controller. The output voltage of the PID controller, after being converted into an analog signal, *U_var_*(*t*), is applied to the other actuator, i.e., Act.2. Here, *U_ref_*(*t*) applied to Act.1 is a reference waveform while *U_var_*(*t*) applied to Act.2 is a corrected waveform that is generated by the PID controller. At any instant, *U_var_*(*t*) is adjusted to make *P*(*t*) to converge to *P_0_*. The error, *e*(*t*), is a measure of the residual tilting of the mirror plate, which will diminish through the closed loop with the PID controller. According to the classic PID control theory, the proportional term produces an output proportional to *e*(*t*) to let *P*(*t*) reach *P*_0_ rapidly, but the steady-state error may still exist, which can be eliminated by the integral term. Moreover, there may be a fluctuation of *e*(*t*) when *P*(*t*) is almost equal to *P*_0_, so the derivative term helps to provide a stable response. The gain values, *K_P_*, *K_I_*, and *K_D_*, are tuned based on a basic model identified via the Matlab toolbox, which are *K_P_* = 0.076478, *K_I_* = 2.121529, *K_D_* = −0.000502; respectively. The sampling frequency is set at 10 kHz. According to the step response in this closed-loop control system, the rise time is 16.2 ms and the settling time is 47.2 ms with the overshoot less than 8%.

**Figure 7:**
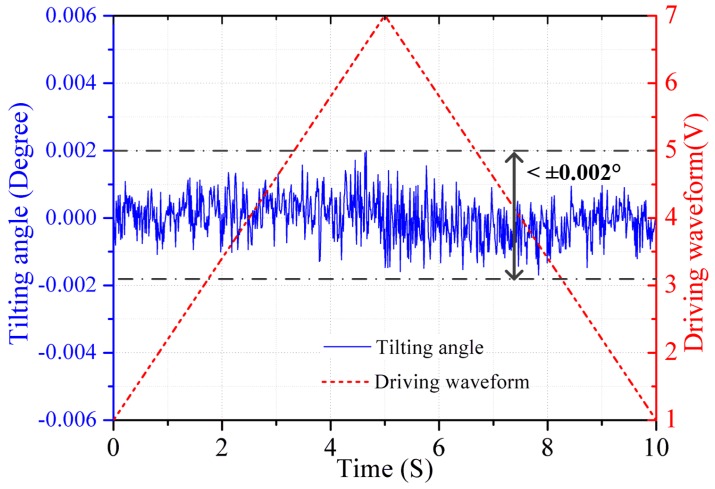
The residual tilt angle versus voltage under closed-loop control.

As *U_var_*(*t*) is adjusted in real time by the PID controller, the laser beam is stabilized at the preset position ***P_0_*** on the PSD, which means the tilting of the mirror plate is compensated effectively during the whole piston motion. Figure 7 shows the residual tilting versus voltage under such a closed-loop control. The tilting angle is significantly reduced down to within ±0.002° in the entire drive voltage range, which meets the mirror tilt requirement of our FTS system. Note that the MEMS mirror has strong nonlinearity and weak response at low voltage as shown in Figure 5a, so the voltage of the reference driving waveform starts from 1 V instead of 0 V to discard the nonlinearity range at low voltage. This leads to only a small loss in the piston scan range.

## 5. FTS Setup & Experiments

**Figure 8:**
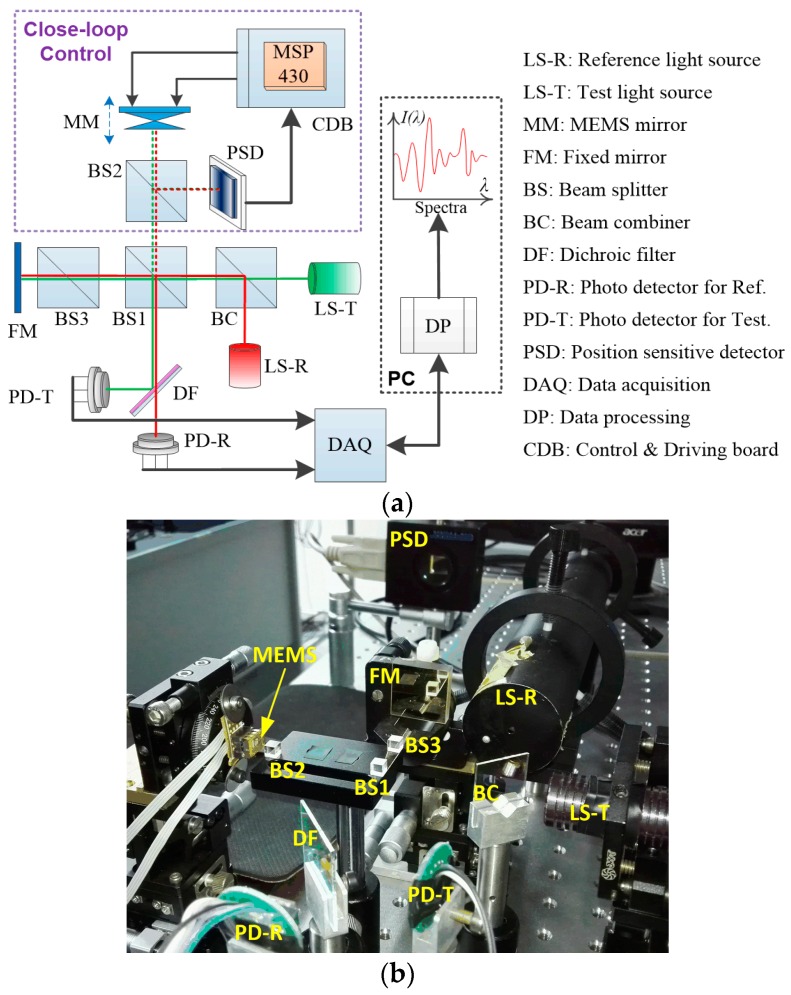
(**a**) The schematic of the FTS system with closed-loop control; (**b**) The experimental setup for demonstration.

An FTS system based on the LSF MEMS mirror with closed-loop control has been built. As illustrated in Figure 8a. The reference light source (LS-R) and the test light source (LS-T) are coupled together by a beam combiner (BC) into the first beam splitter (BS1) that splits the light respectively to an MEMS mirror (MM) and a fixed mirror (FM). In the arm with MM, a portion of the reflected light goes to the PSD for closed-loop control via the second BS (BS2), while the third BS (BS3) is placed in the FM arm as a dispersion compensator. The rest of the light beams reflected back from MM and FM are recombined by BS1 which directs half of the light to the photodetector for the test light (PD-T) and the other half to a dichroic filter (DF) which only lets the reference light pass through to the other photodetector (PD-R). The reference light is used for spectrum calibration to overcome the variable velocity of the MEMS scanning. Just for the purpose of functional demonstration, we take a He-Ne laser (632.8 nm) as the reference light, and a semiconductor green laser (the wavelength may drift around 532 nm) combined with the He-Ne laser as the test light.

Figure 8b shows a picture of the experimental setup constructed corresponding to the layout sketched in Figure 8a. The MCU employed for the closed-loop control is MSP430f169 (Texas Instruments. Inc., Dallas, TX, USA), and one embedded 12-bit analog to digital converter (ADC) is used for acquiring the PSD output signal. The PSD module introduced is PSM 2-10 (On-Trak Photonics, Inc., Irvine, CA, USA) with the active area of 10 mm × 10 mm and resolution of 0.25 µm. Two external 16-bit digital to analog converters (DACs) are used to output the corrected driving waveforms. The photodetectors for both the reference light and the test light are OPT 101 (Texas Instruments Inc.), which covers the spectral range from 200 nm to 1100 nm and integrates the photodiode and a transimpedance amplifier together. A high-speed data acquisition card U2531A (Agilent Tech., Santa Clara, CA, USA) is employed and programmed to obtain and store the temporal interferogram signals of the reference and test light synchronously.

**Figure 9:**
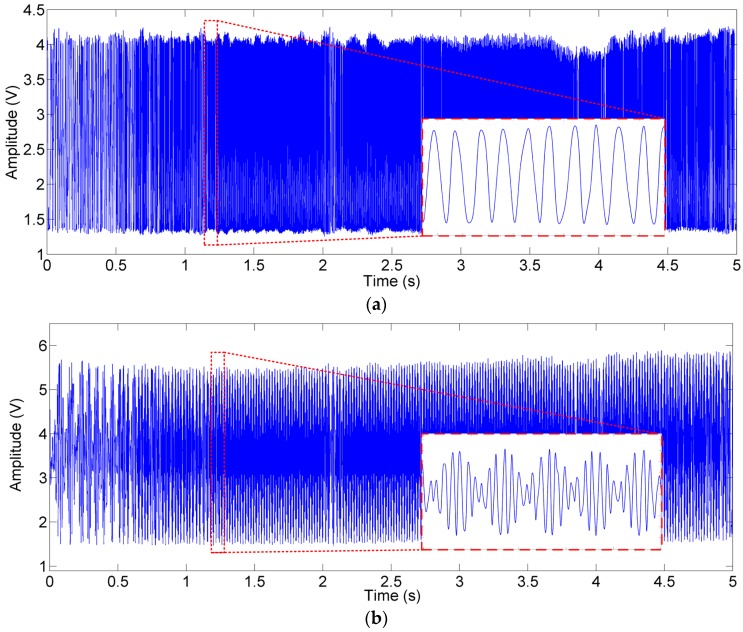
The interferogram signals in time domain: (**a**) the reference light and (**b**) the test light.

After the optical alignment was done, the incident position of the light beam on the PSD was identified when the MEMS mirror was at rest, and the output of the PSD was recorded and then used as the preset value for ***P_0_***. Attributing to the closed–loop PID control, the FTS system reached a stable operation quickly when the MEMS was actuated, and the mirror plate tilting was kept within ±0.002° during the whole scan voltage range from 1 V to 7 V at 0.1 Hz. The interferogram signals of the reference light and the test light, which were generated in one single stroke of the MEMS scanning, were picked up by PD-R and PD-T, as shown in Figure 9a,b, respectively.

**Figure 10:**
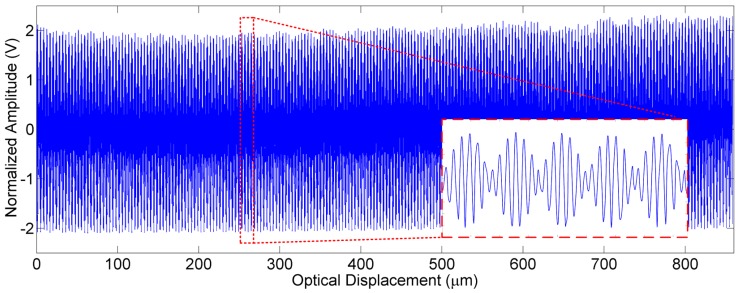
The resampled interferogram signal of the test light in spatial domain.

Before the recovery of the spectra via Fast Fourier Transform (FFT) from the raw interferogram signals, a specific data process [27] was adopted to convert the unevenly sampled time-domain interferogram signals into evenly sampled data in spatial domain, as shown in Figure 10. Therefore, the distortion resulting from the variable scanning velocity of the MEMS was compensated by using the wavelength-known reference light to measure the position of the MEMS mirror plate. 

Meanwhile, the optical path difference (OPD) scanned by the MEMS was accurately calculated based on the reference interferogram signal. According to the experiments, the total OPD reached as much as 860 μm per scan, corresponding to a physical displacement of 430 μm per scan. Compared to the static piston range of 550 μm (0–7 Vdc) shown in Figure 5a, the stable MEMS scan with the tilting angle controlled under ±0.002° reached up to 78% of the full scan capability of the MEMS mirror. The lost 22% scan range mainly resulted from the unused nonlinear region at low driving voltage, the large thermal response time (~190 ms), and the ambient temperature rise due to the continuous Joule heating on the bimorph actuators.

**Figure 11:**
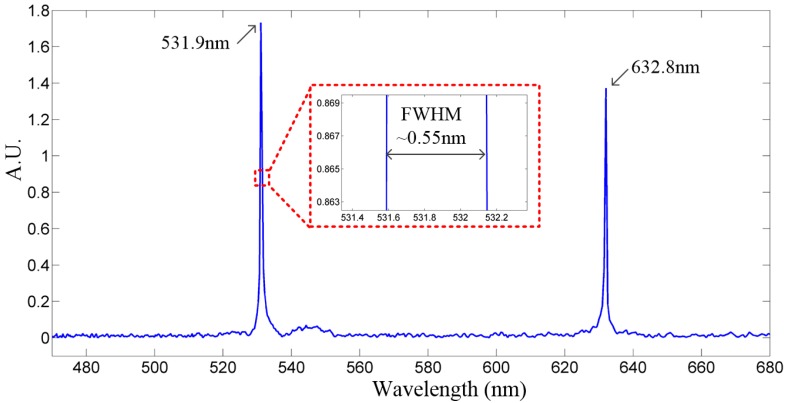
The recovered spectrogram of the test light by FFT.

After FFT and Mertz phase correction, the spectrum of the test light, as shown in Figure 11, was recovered from the spatial-domain interferogram. The spectral peaks of the test light, which is a combination of the green laser and the red laser, were detected. The peak at 632.8 nm accurately indicates the He-Ne red laser, and the 531.9 nm peak corresponds to the semiconductor green laser. The measured FWHM spectral resolution at 531.9 nm is about 0.55 nm, corresponding to 19.4 cm^−1^ in wavenumber. In FTS spectroscopy, the best resolution in wavelength, δ_λ_, is given by Equation (7).

(7) $$\delta_{\lambda }=\frac{\lambda^{2}}{OPD_{\max}}$$

where λ is the central wavelength of the light source, and the *OPD*_max_ is the maximum optical scan range. With an 860 μm OPD, the theoretical resolution is 0.33 nm at 531.9 nm. The difference between the theoretical and experimental resolution may result from the residual tilting, external vibrations, and noises. A performance comparison between the former open-loop control method and the closed-loop control method are briefly summarized as Table 3, which clearly indicates that the closed-loop control method provide a great enhancement to the electrothermal MEMS based FTS system.

## 6. Conclusions

An FTS system enabled by a closed-loop controlled electrothermal MEMS mirror has been demonstrated. Compared to the prior work, the newly implemented closed-loop control method provides a robust solution on minimizing the tilting of the MEMS mirror during the piston scanning. The usable OPD scan range with the tilting angle controlled within ±0.002° is significantly extended up to 860 µm, which accounts for 78% of the full scan capability of the MEMS. Thus, a measured spectral resolution of 0.55 nm at 531.9 nm wavelength is achieved. Although this system currently works in the visible range, it can be easily extended to near infrared (NIR) or even IR if proper optical components are available. Besides, as the allowable tilting angle becomes larger for longer wavelengths, the tilting effects on spectral properties will actually be reduced for the NIR/IR range. In the future, a compact FTS with the MEMS mirror closed-loop controlled will be developed by improving the MEMS packaging and system design.

## Figures and Tables

**Table 1:** Comparison of prior work to this work.

Type	Capable Range	Maximum Tilting	Control Method	Compensated Tilting	Usable Range	Utilization
Wu’s first LSF [23]	620 μm	0.7°	None	N/A	N/A	N/A
Liu’s CCBA [24]	200 μm	0.4	None	N/A	N/A	N/A
Laddered ISC [25]	90 μm	0.25°	None	N/A	N/A	N/A
Wu’s second LSF [26]	1000 μm	2.5°	Optimized Ratio Voltages	0.06°	70 μm	7%
Meshed ISC [27]	145 μm	0.37°	Optimized Ratio Voltages	0.004°	48 μm	33%
Wang’s LSF [28]	650 μm	0.3° (@3.5V)	Open-loop Control	0.002°	225 μm	34%
Present work	**550 μm**	**0.65**°	**Closed-loop Control**	**0.002°**	**430 μm**	**78%**

LSF is lateral-shift-free, CCBA is curled concentric electrothermal bimorph actuator, and ISC is inverted-series-connected.

**Table 2:** The structural parameter of the MEMS device.

	Length	Width	Thickness
Mirror plate:	1.1 mm	1.1 mm	30 µm
Extended bridge beam	1 mm	150 µm	31.7 µm ^1^
Bimorph beam (B_1_,B_2_,B_3_)	150 µm, 300 µm,150 µm	10 µm	2.2 µm
Rigid beam (R_1_,R_2_)	1 mm, 1 mm	160 µm	31.7 µm ^1^

^1^ The extended bridge beams and rigid beams consist of the 30 µm-thick silicon device layer and all of the deposited SiO_2_ layers which are 1.7 µm in total.

**Table 3:** The comparison between the open-loop and closed-loop control.

Performance	Open-Loop Control [28]	Closed-Loop Control
Rising time	190 ms	16.2 ms
Maximum OPD	225 µm	430 µm
Scan range Utilization	34%	78%
Spectral resolution	33 cm^−1^	19.4 cm^−1^
